# Assessing the Quality, Readability, and Acceptability of AI-Generated Information in Plastic and Aesthetic Surgery

**DOI:** 10.7759/cureus.73874

**Published:** 2024-11-17

**Authors:** Muireann Keating, Stephanie M Bollard, Shirley Potter

**Affiliations:** 1 Department of Plastic and Reconstructive Surgery, St James's Hospital, Dublin, IRL; 2 School of Medicine, University College Dublin, Dublin, IRL; 3 Plastic and Reconstructive Surgery, Mater Misericordiae University Hospital, Dublin, IRL

**Keywords:** aesthetic surgery, artificial intelligence, chatbot, medical technology, plastic surgery

## Abstract

Introduction: Within plastic surgery, a patient's most commonly used first point of information before consulting a surgeon is the internet. Free-to-use artificial intelligence (AI) websites like ChatGPT (Generative Pre-trained Transformers) are attractive applications for patient information due to their ability to instantaneously answer almost any query. Although relatively new, ChatGPT is now one of the most popular artificial intelligence conversational software tools. The aim of this study was to evaluate the quality and readability of information given by ChatGPT-4 on key areas in plastic and reconstructive surgery.

Methods: The ten plastic and aesthetic surgery topics with the highest worldwide search volume in the 15 years were identified. These were rephrased into question format to create nine individual questions. These questions were then input into ChatGPT-4. The response quality was assessed using the DISCERN. The readability and grade reading level of the responses were calculated using the Flesch-Kincaid Reading Ease Index and Coleman-Liau Index. Twelve physicians working in a plastic and reconstructive surgery unit were asked to rate the clarity and accuracy of the answers on a scale of 1-10 and state ‘yes or no’ if they would share the generated response with a patient.

Results: All answers were scored as poor or very poor according to the DISCERN tool. The mean DISCERN score for all questions was 34. The responses also scored low in readability and understandability. The mean FKRE index was 33.6, and the CL index was 15.6. Clinicians working in plastics and reconstructive surgery rated the questions well in clarity and accuracy. The mean clarity score was 7.38, and the accuracy score was 7.4.

Conclusion: This study found that according to validated quality assessment tools, ChatGPT-4 produced low-quality information when asked about popular queries relating to plastic and aesthetic surgery. Furthermore, the information produced was pitched at a high reading level. However, the responses were still rated well in clarity and accuracy, according to clinicians working in plastic surgery. Although improvements need to be made, this study suggests that language models such as ChatGPT could be a useful starting point when developing written health information. With the expansion of AI, improvements in content quality are anticipated.

## Introduction

Public interest in plastics and aesthetic surgery is increasing. The American Society of Plastic Surgeons (ASPS) recently announced a 19% increase in cosmetic surgery procedures since 2019 [1]. The procedures with the largest increase include abdominoplasty (38% increase), breast reduction (54% increase), and liposuction (23% increase). This rise in interest is also evident in a 100% increase in Google search of ‘plastic surgery’ between December 2019 and January 2021 (data source: Google Trends). The Internet is a well-known tool for patient information, with 95% using the Internet as a source of information prior to consulting a plastic surgeon. [2]. The ASPS, in their annual procedural statistics, cites the rise of the internet and social media as one of the reasons for this recent uplift [1].

Patients will often do their own research prior to committing to a surgical procedure [3]. However, the quality and accuracy of medical information available on the internet is a constant concern [4]. Thousands of instantly available articles, blogs, and social media videos are now produced with a simple ‘Google search’. It is important that patients can access high-quality resources that suit their level of understanding and reading comprehension. There are, of course, high-quality, vetted patient resources available freely on professional websites, like the Aesthetic Society and the British Association of Plastic Reconstructive and Aesthetic Surgeons, producing online patient resources on popular aesthetic procedures [5,6]. However, not all patients are aware of the existence of these professional resources and are able to navigate the websites to find the answers to their specific questions. Furthermore, these resources are often buried beneath thousands of potentially inaccurate search results.

The rise of free-to-use public Artificial Intelligence (AI) programmes has added another platform where patients can search for guidance. Artificial intelligence refers to the use of computer technology to simulate and approximate human critical thinking [7]. ChatGPT is a freely available online AI tool, known as a large language model, and was developed by OpenAI. It is based on the Generative Pretrained Transformer (GPT) architecture and has been trained on a large volume of text data. Its purpose is ‘to generate human-like responses to natural language inputs, such as questions or statements about any topic’. ChatGPT was developed to be an AI language model that was capable of assisting in various tasks such as data analysis, translation, and text generation.

AI tools like ChatGPT are attractive applications for patient information to both clinicians and patients due to their ability to instantaneously answer almost any query. This rise in popularity may see patients using these tools to obtain further information on surgical procedures and their associated risks. As this is a relatively new information resource, the safety of the information produced still remains unknown. The aim of this study was to evaluate the quality and readability of information given by ChatGPT4 on key areas in plastic and reconstructive surgery.

## Materials and methods

Using Google Trends, the ten Plastic and Aesthetic Surgery topics with the highest worldwide search volume in the 15 years were identified. Topics with the highest search volume related to plastic and aesthetic surgery were rephrased into question format to create nine individual questions (Table 1). These questions were then input into ChatGPT4 to create an interactive dialogue. Each question was entered as a new prompt, and the first response produced was recorded. The 'regenerate response’ feature was not used. All Chat GPT4-generated responses were documented for analysis.

**Table 1:** Table of 9 questions developed from most commonly asked plastic surgery queries from Google trends.

Question:
What is plastic surgery?
What is reconstructive surgery?
What is the difference between plastic and reconstructive surgery?
What is a rhinoplasty?
Are rhinoplasties safe?
What are the complications of abdominoplasties?
What is breast augmentation surgery?
What are the reconstructive surgery options after breast cancer?
What is capsular contracture?

Quality analysis

The responses generated by ChatGPT4 for each question were evaluated and rated by the author using the DISCERN instrument [8]. The DISCERN questionnaire is a validated and reliable instrument for analyzing written consumer health information [8]. The first eight questions focus on the reliability of the information; the next seven questions focus on the quality of treatment information; and the final questions look at overall quality. Each question is rated with an ordinal Likert scale from 1 to 5 (no to yes). The tool can give a maximum score of 80. Higher scores are associated with higher quality [8].

Readability analysis

The answer readability was assessed using the Flesch-Kincaid Reading Ease Index (FKRE Index). The FKRE index ranges from 0 (unreadable) to 100 (very easy to read). An FKRE index score of greater than 60 is easily understood by 13- to 15-year-old students and is the recommended level for public information [9]. The Coleman-Liau Index was calculated for each answer to assess the understandability and US-grade reading level of the answers. A standard aim when writing text for public use is for around 7th-8th grade in the US grade level system (12-14 years), which equates to a score of 8 or less [10].

Clarity and accuracy

A total of 12 physicians, six consultants, and six trainees working in a Plastic and Reconstructive Surgery unit were asked to rate the clarity and accuracy of the answers on a scale of 1-10, with 10 being most accurate/clear and one being least accurate/clear. They were also asked to state ‘yes or no’ if they would share the generated response with a patient. For the purpose of rating, accuracy was defined as ‘the answer is conforming exactly to truth or to a standard’, and clarity was defined as ‘content that is easy to read and understand’. Answers with higher scores were assessed as being of higher accuracy and/or clarity.

Statistical analysis

Data analysis was performed using Microsoft Excel (Redmond, USA). Summary statistics are provided. Analysis of variance was used to compare means for continuous variables of interest when appropriate.

## Results

Quality analysis

**Figure 1:**
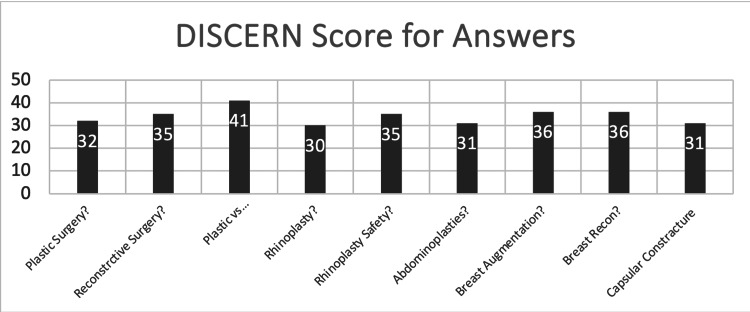
Bar chart of DISCERN scores for the ChatGPT4-generated responses for each individual question.

All answers were scored as poor or very poor according to the DISCERN tool. The mean DISCERN score for all questions was 34. Figure 1 shows the DISCERN score for each question. 

Readability

The responses also scored low in readability and understandability, with no answer meeting the recommended levels of public written information. The mean FKRE index was 33.6 and the CL index was 15.6. Table 2 shows the FKRE index and CL index for each question.

**Table 2:** Table breakdown of the FKRE Index and CL Index for ChatGPT4 responses for each question.

Question	FKRE Index	CL Index
What is plastic surgery?	28.6	14.4
What is reconstructive surgery?	23.7	17.7
Plastic vs. reconstructive?	19.7	19.1
What is a rhinoplasty?	42.1	13.4
Rhinoplasty safety?	32.9	16
Abdominoplasty?	31.2	15.6
Breast augmentation?	37.3	15.5
Breast reconstruction?	44	14.2
Capsular contracture?	43.1	14.8

Clarity and accuracy

**Figure 2:**
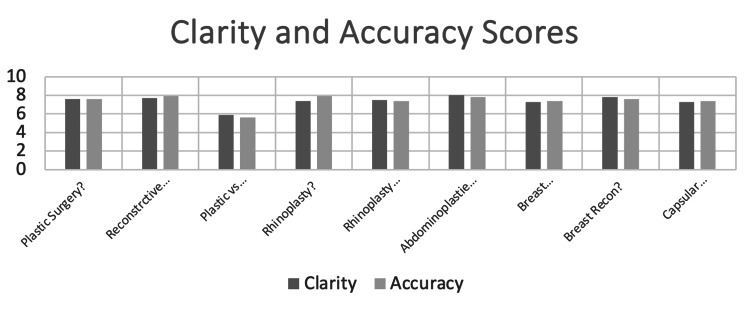
Bar chart of the average clarity and accuracy score ratings for ChatGPT4 responses to each question.

Clinicians working in plastics and reconstructive surgery rated the questions well in clarity and accuracy. Figure 2 shows the clarity and accuracy score for each question. The mean clarity score was 7.38 and the accuracy score was 7.4.

**Figure 3:**
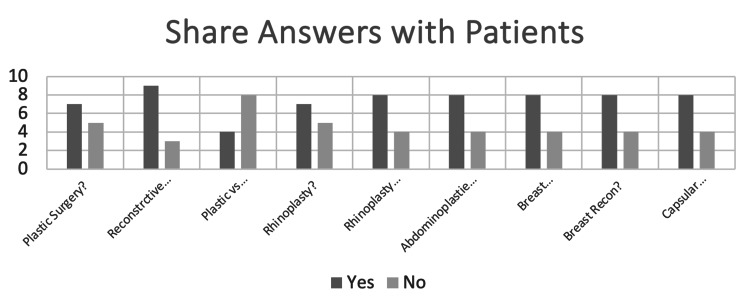
Bar chart of the number of physicians working in plastic surgery who would be happy to share ChatGPT4-generated responses for each question with a patient.

When asked, the group of clinicians reported they would be happy to share 62% (range 34% to 75%) of the ChatGPT4-generated responses with a patient (Figure 3).

## Discussion

The quality of online health information plays a crucial role in providing safe, accurate information to patients. A major finding from this study was the low quality of the DISCERN score responses produced by ChatGPT4. There are a number of potential reasons for this. First, ChatGPT does not cite formal sources or references for its responses [11], and when references are requested, they may often be fake. It creates answers to users' questions through an interactive interface. ChatGPT is based on the GPT (Generative Pre-trained Transformer) architecture [12]. First introduced by OpenAI in 2018, this software architecture is a type of transformer-based neural network that is trained on large amounts of text data and that can then generate coherent and grammatically correct text [12,13]. Appropriate citation and sourcing is a vital part of quality assessment in healthcare information that ChatGPT lacks. Next, there is an argument that traditional validated quality assessment tools, like DISCERN, are not suitable for assessing the information produced by ChatGPT. DISCERN, the tool used for our quality assessment, was developed in 1999 by an expert panel in consumer health information to judge the quality of written information about different treatment choices [8]. The written information it targets is health information leaflets and websites. Although ChatGPT presents the information in written format, it is not the same as these traditional ‘written health information’ resources. Tools like DISCERN focus on information sources and evaluate information quality based on comprehensive treatment descriptions [8,14]. AI chatbots, like ChatGPT, are designed to provide specific answers based on conversation-like interactions with the user. Similarly, the JAMA benchmark criteria is a popular method for assessing information quality. It comprises 4 standards to assess transparency and reliability, including authorship, attribution, disclosure, and currency, with each standard scoring 1 point for a maximum total of 4 [15]. Again, as ChatGPT does not site references unless prompted, all responses automatically score a zero in the JAMA benchmark. This suggests that different tools should be used when assessing the quality of AI-produced information.

A high reading level was required to comprehend the responses produced. The average FKRE index of the answers was 33.6, with the recommended being 60. Higher reading level requirements are an ongoing issue with online written health information [16]. High-quality health information written by vetted resources is consistently pitched at a higher reading level. This poses a potential risk of spreading misinformation to patients due to a lack of understanding. Furthermore, misunderstanding may prompt patients to seek health advice from poorer quality, inaccurate resources. A change in reading level is something that can be simply altered by AI software by changing the way the question is asked. An advantage of AI-generated responses is their ability to specify the age or reading level of the response through appropriate prompting. For example, by asking Chat GPT3 to ‘Explain what is plastic surgery at a grade 5 reading level’, one gets a different response:

“Plastic surgery is a medical practice where doctors use operations to change or enhance the way someone looks. It can involve fixing things like noses or ears or making the skin smoother. People might choose plastic surgery to improve their appearance or fix something they don't like. It's a specialized type of medical treatment that helps people feel better about how they look.”

This response scores a 56.6 FKRE index and an 11 CL CL index. Although both scores are still not within the recommended range, it is a significant improvement to the initial response. The use of AI tools like ChatGPT has the potential to be a useful resource for producing high-quality health information at varying targeted reading levels.

Although the responses were graded as poor quality by the DISCERN tool, they were overall rated well in clarity and accuracy by clinicians working in plastic and reconstructive surgery. The responses given by ChatGPT3 were rated on average 7.38 in clarity and 7.4 in accuracy. This clarity and accuracy assessment tool was ultimately subjective but gives an insight into clinician acceptability of AI-generated medical information. Clinicians' acceptance of AI-generated medical information, as well as the role of AI in healthcare, is an area of increasing interest with the rise of AI. Previous studies rating the accuracy of physician ratings generated by AI have found similar results. A study by Johnson et al., which looked at AI-generated medical information across a variety of specialties, found that clinicians rated the majority of information as “nearly all correct, or "correct," with a mean accuracy score of 4.8 (out of 6) [17].

Fear of misinformation and unsafe advice is an ongoing fear when reviewing the role of online resources as points of patient information. It is well documented in the literature that patients often access information online that is inaccurate and of poor quality [4,18]. When reviewing where AI programmes, like ChatGPT, get their baseline information from, this fear still remains. ChatGPT is trained on a large body of text from a variety of online resources [19]. This includes scientific journals and public health information websites. However, it also includes webpages like Wikipedia, forums, and news websites-resources that are all at risk of misinformation and inaccurate data. A number of studies have shown that ChatGPT can present factually incorrect information with factitious references when asked to produce healthcare-related literature [20-22]. A study by Ariyaratne et al. compared ChatGPT-generated articles with human-written articles [21]. The most striking finding of the study was ChatGPTs’ ability to produce coherent research articles that closely resemble authentic research. However, these articles contained factually incorrect information with fake resources. This is a danger because, to a patient, a well-presented article with what seems like appropriate references may be taken as factual. Furthermore, conversational AI models, like ChatGPT, often struggle to maintain the context of conversation [19]. This means it can misunderstand a question and give a ‘wrong’ answer. This occurs most commonly with ‘ambiguous queries’, highlighting that AI responses are highly dependent on the way the questions are asked and small differences in phrasing can alter its response.

Patient selection and establishing achievable expectations play crucial roles in the consultation process for aesthetic surgery. These are areas that are explored from the initial consultation with a plastic surgeon and are an important deciding factor if a patient is suitable for their desired procedure. A ‘ChatGPT consultation’ overlooks this key area. There is a well-documented link between aesthetic surgery, psychology, and social environment, with appropriate patient selection playing a key role in both physical and psychological postoperative complications [23]. The majority of cases of patient dissatisfaction in aesthetic surgery are based on failures of communication and patient selection criteria rather than technical errors [24]. If a patient were to wholly make a decision on a procedure based on internet and AI-generated information, the surgeon potentially faces a more challenging situation in establishing realistic expectations from the outset.

Since this study was performed, there have been further updates and paid versions of ChatGPT and other AI software, including medical-specific programmes that make promises of higher quality. ChatGPT4 was chosen for this study because it is free to use and one of the more popular software available, making it the most likely programme a patient would use. Unlike a specifically designed medical chatbot, ChatGPT has not been fully trained on the specific datasets created by medical professionals [25]. Furthermore, its database is not yet fully up to date, with the knowledge cut-off being September 2021 at the time of this study. This increases the risk of both lower-quality and inaccurate information. However, this is an area that will see significant change in the near future. A fundamental aspect of AI is its ability to learn from use [19]. ChatGPT uses reinforcement learning from human feedback (RLHF) to fine-tune responses. In simple terms, it takes feedback from user answers and the regenerate response button to build information and phrasing. Launched in 2022, ChatGPT is a software model that is still in its infancy. However, it has already undergone significant changes and improvements. As time goes on, we can only expect further developments in this area, bringing with it higher-quality information.

This study is limited by the number of participants. This was a single-centre study conducted in one plastic and reconstructive unit attached to a university hospital. Furthermore, as discussed above, this study is limited by the rate of expansion of AI software tools like ChatGPT. ChatGPT 4's cutoff for up-to-date information is April 2023. ChatGPT is only one of many free-to-use AI software models that patients may use to obtain such information.

## Conclusions

The incorporation of AI into the healthcare industry is ultimately inevitable. It is our role as plastic surgeons to guide patients on how to use these tools correctly. This study highlighted that although ChatGPT responses to commonly asked questions in plastic and aesthetic surgery are of lower quality, we currently do not have the tools to formally assess this format of information in an evidence-based matter. As the area grows, we expect to see not only the production of higher-quality information but also specific AI tools to evaluate it. Furthermore, AI tools like ChatGPT are potentially useful sources to improve medical written information’s readability and understandability. Overall, the AI-generated responses were accepted by clinicians working in plastic and aesthetic surgery, suggesting it could serve as a useful baseline tool when advising patients in the clinic. Nevertheless, ChatGPT is not a replacement for clinician-led consultations. Practical experience, setting realistic expectations, and interpersonal skills, all remain vital aspects of information giving and the overall consultation experience skills yet to be replicated by a ‘chatbot’.
